# Backyard evolutionary biology: Investigating local flowers brings learning to life

**DOI:** 10.1002/ece3.7199

**Published:** 2021-01-26

**Authors:** Abha Ahuja

**Affiliations:** ^1^ Natural Sciences Minerva Schools at Keck Graduate Institute San Francisco CA USA

**Keywords:** Evo‐devo, flower whorls, Inquiry‐based learning, online instruction

## Abstract

Inquiry‐based learning allows students to actively engage in and appreciate the process of science. As college courses transition to online instruction in response to COVID‐19, incorporating inquiry‐based learning is all the more essential for student engagement. However, with the cancelation of in‐person laboratory courses, implementing inquiry can prove challenging for instructors. Here, I describe a case that exemplifies a strategy for inquiry‐based learning and can be adapted for use in various course modalities, from traditional face‐to‐face laboratory courses to asynchronous and synchronous online courses. I detail an assignment where students explore the developmental basis of morphological evolution. Flowers offer an excellent example to address this concept and are easy for students to access and describe. Students research local flowering plants, collect and dissect flower specimens to determine their whorl patterns, and generate hypotheses to explain the developmental genetic basis of the patterns identified. This task allows students to apply their scientific thinking skills, conduct guided exploration in nature, and connect their understanding of the developmental basis of evolutionary change to everyday life. Incorporating inquiry using readily available, tangible, tractable real‐world examples represents a pragmatic and effective model that can be applied in a variety of disciplines during and beyond COVID‐19.

## Graphical abstract

Incorporating inquiry‐based learning is essential for student engagement and developing scientific thinking skills. I describe an assignment where students conduct guided inquiry of their local flowering plants and generate hypotheses to explain the developmental genetic basis of the whorl patterns observed. Adopting the model exemplified by this assignment and using examples that can be readily accessed for hands‐on scientific inquiry can be very effective during COVID‐19 and after.

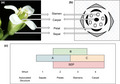

## 1 INTRODUCTION

For students to understand and appreciate the scientific method, they need opportunities to actively engage in the process of investigation and discovery. Inquiry‐based learning is a pedagogical approach that emphasizes investigative projects that mirror the scientific process (Gehring & Eastman, 2008; Handelsman, 2004). Students answer questions and solve problems by gathering and interpreting data. This approach is associated with improved student analytical and critical thinking skills, better retention of course content, and increased engagement (Kolkhorst et al., 2001, DiPasquale et al., 2003; Freeman et al., 2014; Handelsman, 2004). Typically, inquiry‐based learning is incorporated in science laboratory courses where students design experiments to collect and analyze data. With the pivot to remote instruction and cancelation of in‐person laboratory courses, instructors must incorporate inquiry‐based learning in new and creative ways that are robust to constraints posed by COVID‐19.

Here, I describe a course assignment that provides a practical model for how instructors might incorporate hands‐on, inquiry‐based learning during COVID‐19 posed restrictions and beyond. In this model, instructors identify tangible, everyday examples of fundamental scientific concepts and utilize materials that are readily available and accessible for students. This model can be readily adapted to address a variety of biology concepts in different learning modalities, including synchronous and asynchronous remote learning. First, I identified and defined a fundamental concept to target in the assignment: An essential aspect of teaching evolution and morphological change is understanding underlying developmental mechanisms (Perez et al., 2013). However, Hiatt et al. (2013) found that evolutionary biology curricula typically address development superficially and that students lack adequate understanding of foundational concepts from developmental, cell, and molecular biology. This is also reflected in the fact that the literature on teaching and learning of Evolutionary Developmental Biology (evo‐devo) is sparse compared to subfields such as natural selection and phylogenetics (Ziadie & Andrews, 2018). Therefore, I sought to design curriculum to address this gap.

**FIGURE 1:**
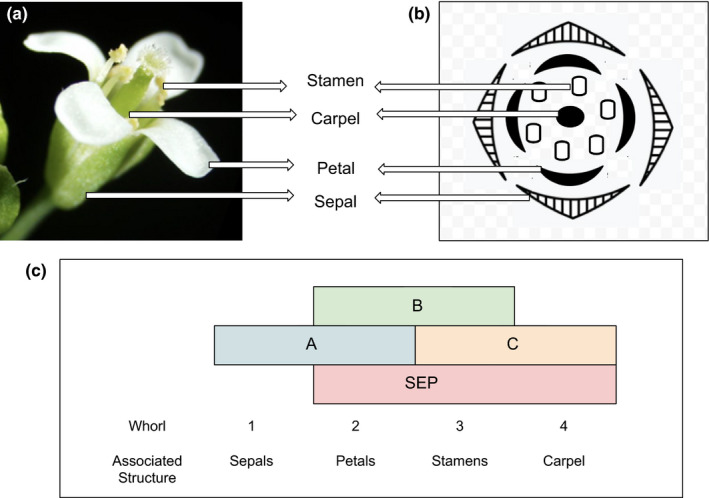
*Arabidopsis thaliana* (a) wild‐type flower (adapted from Sui‐Setz, 2020) and (b) floral diagram showing the four concentric whorls. (c) Expression patterns of ABC genes specify whorl identity: Expression of A alone is associated with sepal identity in the outermost whorl. A combination of A, B, and SEP is associated with petals in the second whorl and B, C, and SEP with stamens in the third whorl. Expression of C and SEP genes is associated with carpel formation in the fourth whorl

**FIGURE 2:**
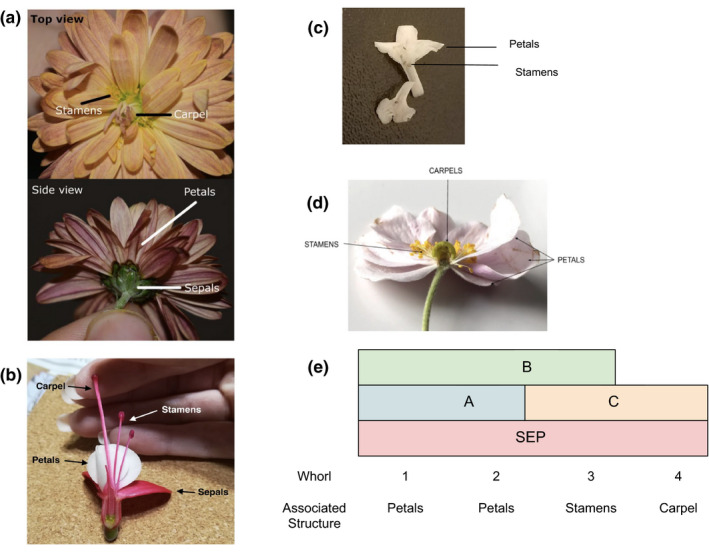
Selected examples from students' submissions showing variation in floral form (a) *Chrysanthemum morifolium*, (b) *Fuchsia magellanica*, (c) *Viburnum farreri*, and (d) *Anemone hupehensis*. (e) Changes in expression patterns of ABC genes can explain variation in whorl patterns: an illustrative example showing how increase in the spatial domain of expression of B and SEP class genes and a concomitant reduction in the spatial domain of gene A expression can explain the loss of sepals and double‐petal phenotype

Next, I identified a compelling and readily available example of this concept: flower variation. Flowers are amazingly diverse in their color, size, shape, and smell. Flower whorl patterns are a particularly well‐studied aspect of flower morphology. The model plant *Arabidopsis's* typical flower comprises four concentric whorls: sepals, petals, stamens, and carpels (Figure 1). The simplified ABC model explains the developmental genetic basis of whorl development. The expression patterns of four main classes of homeotic transcription factor encoding genes called *A*, *B*, *C*, and *Sepallata* (*SEP*) genes are associated with the identity of specific whorls in *Arabidopsis* (Figure 1) (Irish, 2017). Gene *A* products are associated with the formation of sepals in the outermost whorl. In the second concentric whorl, products of *A*, *B,* and *SEP* genes promote petal formation while expression of *B*, *C,* and *SEP* genes in the third whorl promotes stamens. Finally, *C* and *SEP* genes promote carpel formation in the innermost whorl. The number and arrangement of whorls vary widely. Changes in ABC genes’ spatial expression patterns are associated with the changes in whorl patterns in different floral species (Figure 2). Therefore, flower whorl variation provides an excellent example to illustrate how changes in gene expression during development can generate variation between species.

I developed an assignment where students research their local flowering plants by collecting and dissecting flower specimens to describe how the whorl patterns differ from that in Arabidopsis. Observing and documenting flower morphology is straightforward and does not require extensive instructor or student training. Whorl patterns can be determined with the naked eye for most flowers, so it is a cost‐effective activity with little need for equipment or supplies. Students then generate and justify a hypothesis to explain the developmental genetic basis of the whorl patterns observed; that is, students explain how changes in the spatial expression patterns of ABC genes might lead to the changes in whorl patterns seen in their flower as compared to *Arabidopsis*. These tasks sharpen students’ scientific skills and connect their learning to the natural world around them.

## 2 ASSIGNMENT DESCRIPTION

The assignment I describe was implemented in an upper‐level genetics course at the Minerva Schools at Keck Graduate Institute (California, USA). Minerva is a primarily undergraduate institution where students are in a global rotation program, living in different cities during their studies. We teach all courses in virtual classrooms via video conferencing on our proprietary platform using active learning pedagogy; therefore, all our courses and curriculum, including this assignment, have been designed and optimized for an online format. Each course includes a Location‐Based Assignment (LBA) that engages students with their residence location and allows them to connect their coursework to the local context.

First, I tasked students with conducting a literature search on local flowering plants of the region they reside in and determining specific sites to access selected plants in their flowering season. Under ideal conditions, students are encouraged to collect local flowers from nearby parks, forests, or nature reserves. Students could use applications such as iNaturalist to identify plant species in their backyards (iNaturalist, n.d.). However, as I discuss below, I suggest adding sites such as botanical gardens, nurseries, greenhouses, and even house plants in the event that local flowering plants are not readily accessible. Students then visit their selected sites individually or in small groups to collect flower specimens. I asked students to ideally find an intact flower fallen to the ground, or obtain verbal permission to pluck a flower if needed. Students dissect the flower to determine its whorl patterns and record images of the intact and dissected flowers. I directed students to open access resources (e.g., How to Draw a Floral Diagram (With Diagram), 2015) to self‐learn how to dissect the flower and draw a floral diagram.

In their submission, students were asked to describe their selected plant, with a focus on adaptations that allow it to grow in the regional conditions such as climate, type of soil, and pollinator species. I also encouraged students to note whether the flower has any cultural significance (e.g., wedding flower or other celebrations). Students reported the flower whorl pattern, including images of the flower and floral diagrams (Figure 2). They proposed and justified a hypothesis to explain how changes in ABC genes' expression patterns might lead to the whorl patterns seen in their flower compared to *Arabidopsis*. I assigned students chapters from their course textbook (Brooker, 2012) and open‐source articles (Irish, 2017; Krizek & Fletcher, 2005) as resources for their research.

## 3 OUTCOMES

The assignment was successful in allowing students to connect their coursework to everyday life. In the end of term course survey, 83% of respondents agreed or strongly agreed with the statement “*The (Location Based) Assignment helped me apply the course learning outcomes in real life*.*”* Selected student comments included “*The (Location Based) Assignment was really great and fun. It was nice seeing how genetics applied to real life*” and “*Really enjoyed the hands‐on aspect and looking at flowers.”*

In a class of seventeen students, thirteen unique flowers were selected. The following student comment from the end of course survey illustrates an important consideration for specimen collection “I really liked the assignment but I did get a bit disheartened when I was trying to find a flower in Berlin in December because there's not a lot of things flowering at that time of year. It did lead to some interesting research, though, *because* most things aren't flow(er)ing in December and this plant was.” This is particularly relevant in light of restrictions due to COVID‐19. While students may still be able to explore open natural areas, access to sites like botanical gardens or greenhouses will likely remain limited. Also, depending on the weather at the time of the year that the assignment is introduced, there may be fewer flowering species in students' regions. Instructors can expand students’ options for site visits to include local florists, grocery stores, or house plants to research commercially available varieties.

Most students accurately determined flower whorl patterns and effectively generated hypotheses based on the existing model of spatial patterns of expression of ABC genes. The assignment prompt was open‐ended in that we did not explicitly prompt students to provide in‐depth explanations for the molecular mechanisms underlying the changes in spatial domain of gene expression. When students did give more detailed explanations for the molecular basis of changes in gene expression, these provided critical insight into their understanding. For instance, in the scenario where a class of genes are expressed in an additional whorl, as illustrated in Figure 2 panel (e), one student proposed that gene duplication is a possible mechanism underlying the increase in the spatial domain of gene expression. This explanation fails to account for the role of cis‐ and transregulatory elements: Changes in gene expression can occur due to small changes in regulatory elements. This has been previously documented as a commonly held student misunderstanding (Hiatt et al., 2013). Another common student challenge in developmental biology is recognizing the temporal aspects of development (Hardin, 2008). Indeed, students' explanations did not effectively distinguish between the timing of expression of genes during bud development and adult flowers.

In courses where students are expected to have background knowledge of the molecular mechanisms, I recommend that instructors give more detailed instructions asking students to elaborate on the underlying molecular basis. Explanations of students' reasoning can yield critical insights into student learning and understanding (Krontiris‐Litowitz, 2009; Russ et al., 2009).

## 4 CONCLUSION

The assignment described here captures key components of inquiry‐based learning: Students explore the literature to select and describe suitable local species for analysis. Next, they engage in data collection to determine flower whorl patterns, and pose a scientific question based on these data: How might the specific whorl pattern arise during development? Finally, they draw on prior scientific models of the developmental genetic basis of flower whorl determination to formulate and justify an explanation for their selected species’ observed whorl patterns**.** This assignment can be adopted as written in evolutionary biology, genetics, and developmental biology courses as an asynchronous course assessment, ideally with opportunities for scaffolded formative feedback from the instructor. It can also be modified for face‐to‐face or virtual synchronous meetings or laboratory courses. For instance, students can work in small groups and engage in peer instruction to compare whorl patterns and genetic models in curated specimens. Investigations of floral variation can also be adapted for use more widely in ecology and biodiversity courses. More broadly, backyard science has been shown to be an effective approach to provide students meaningful research experiences in a remote setting (McKinnon, 2020; Rahn, 2020), and projects involving observations of plant life are particularly suitable for student‐led data collection (Creech & Shriner, 2020; Long & Wyse, 2012).

Instructors in any discipline wishing to incorporate inquiry into their practice can adopt the model developed here: Identify everyday, readily available examples that illustrate fundamental concepts and design tasks around the example that enable students to conduct research, draw conclusions, and communicate their findings. specific tasks around the example that enable students to conduct research, draw conclusions, and communicate their findings. Inquiry‐based activities are all the more relevant and necessary in this time of distance learning. Feasibility, insufficient time, and logistical constraints can pose significant barriers for incorporating inquiry in curricula in the best of times (Brownell and Tanner, 2012, Borcherding et al., 2019). The model that we describe allows instructors to infuse inquiry practically during COVID‐19 and beyond.

## CONFLICT OF INTEREST

The author declares no conflict of interest.

## AUTHOR CONTRIBUTIONS

**Abha Ahuja:** Conceptualization (equal); investigation (equal); methodology (equal); project administration (equal); writing–original draft (equal); writing–review and editing (equal).

## Data Availability

The submission does not include any supporting information files.
